# Cholinergic Projections From the Pedunculopontine Tegmental Nucleus Contact Excitatory and Inhibitory Neurons in the Inferior Colliculus

**DOI:** 10.3389/fncir.2020.00043

**Published:** 2020-07-16

**Authors:** William A. Noftz, Nichole L. Beebe, Jeffrey G. Mellott, Brett R. Schofield

**Affiliations:** ^1^School of Biomedical Sciences, Kent State University, Kent, OH, United States; ^2^Department of Anatomy and Neurobiology, Hearing Research Group, Northeast Ohio Medical University, Rootstown, OH, United States

**Keywords:** acetylcholine, auditory, choline acetyltransferase, midbrain, viral tracing, hearing, neuromodulation, arousal

## Abstract

The inferior colliculus processes nearly all ascending auditory information. Most collicular cells respond to sound, and for a majority of these cells, the responses can be modulated by acetylcholine (ACh). The cholinergic effects are varied and, for the most part, the underlying mechanisms are unknown. The major source of cholinergic input to the inferior colliculus is the pedunculopontine tegmental nucleus (PPT), part of the pontomesencephalic tegmentum known for projections to the thalamus and roles in arousal and the sleep-wake cycle. Characterization of PPT inputs to the inferior colliculus has been complicated by the mixed neurotransmitter population within the PPT. Using selective viral-tract tracing techniques in a ChAT-Cre Long Evans rat, the present study characterizes the distribution and targets of cholinergic projections from PPT to the inferior colliculus. Following the deposit of viral vector in one PPT, cholinergic axons studded with boutons were present bilaterally in the inferior colliculus, with the greater density of axons and boutons ipsilateral to the injection site. On both sides, cholinergic axons were present throughout the inferior colliculus, distributing boutons to the central nucleus, lateral cortex, and dorsal cortex. In each inferior colliculus (IC) subdivision, the cholinergic PPT axons appear to contact both GABAergic and glutamatergic neurons. These findings suggest cholinergic projections from the PPT have a widespread influence over the IC, likely affecting many aspects of midbrain auditory processing. Moreover, the effects are likely to be mediated by direct cholinergic actions on both excitatory and inhibitory circuits in the inferior colliculus.

## Introduction

Acetylcholine (ACh) plays a wide range of roles in normal auditory function, exerting influence from the cochlea to the auditory cortex. In the forebrain, ACh contributes to memory, learning, and attention (Hasselmo and Sarter, 2011; Leach et al., 2013). In the thalamus, ACh differentially influences the efficacy of inputs to a cell, affecting the gating of information flow and possibly biasing a cell toward top-down vs. bottom-up modulation (Sottile et al., 2017). At many levels of the auditory system, ACh can increase spontaneous activity and excitability of auditory neurons and can alter tuning profiles of cells (Farley et al., 1983; Sarter and Bruno, 1997; Ji et al., 2001; Metherate, 2011; Suga, 2012). Finally, ACh is a primary neurotransmitter in the olivocochlear system and plays a critical role in the cochlear amplifier (Dallos et al., 1996; Ryugo et al., 2011). Although much of the previous research on ACh in the auditory system has been done at the levels of forebrain and cochlea, the evidence is accumulating for widespread and varied effects of ACh in the inferior colliculus (IC), a midbrain hub for both ascending and descending auditory pathways (Winer and Schreiner, 2005; Schofield and Beebe, 2019).

Application of ACh to the IC affects the responses to auditory stimuli of a majority of IC neurons (Watanabe and Simada, 1973; Farley et al., 1983; Habbicht and Vater, 1996). Such effects can modify temporal processing and forward masking (Felix et al., 2019). Supporting the idea of widespread effects of ACh in the IC, both nicotinic and muscarinic ACh receptors are present throughout the IC, as is acetylcholinesterase, the enzyme that degrades ACh (Shute and Lewis, 1967; Cortes et al., 1984; Glendenning and Baker, 1988; Henderson and Sherriff, 1991; Happe and Morley, 2004). This is relevant because the physiological studies described above focused on cells in the central nucleus of the IC (ICc), the main lemniscal division of the IC. The dorsal cortex (ICd) and the lateral cortex (IClc) are extralemniscal subdivisions that give rise to parallel ascending pathways that terminate in different parts of the thalamus and serve a variety of functions. These three subdivisions vary concerning cholinergic innervation; in fact, the extralemniscal divisions typically exhibit the highest levels of cholinergic receptors. Staining for β4 nicotinic cholinergic receptor subunits is heaviest in layer 2 of the IClc, with moderate expression in the ICc and less in the ICd (Gahring et al., 2004). These receptors have recently been reported to aid in the modulation of spike timing and forward masking in the IC (Felix et al., 2019). The IClc has also been noted for its comparatively high levels of the α7 nicotinic receptor subunit and high levels of acetylcholinesterase (Happe and Morley, 2004; Dillingham et al., 2017). Muscarinic receptors also stained differentially in the IC, with an expression of m2 receptors highest in the IClc and ICd and less so in the ICc (Hamada et al., 2010). All of this points to a diverse effect of ACh onto several different regions of IC which are known to participate in different parallel ascending auditory and multisensory pathways (Calford and Aitkin, 1983; Rouiller, 1997; Mellott et al., 2014).

Despite the numerous studies of cholinergic receptors in the IC, there is very little information about the identity of IC cells that are directly targeted by the cholinergic inputs. Neurons of the IC are glutamatergic or GABAergic, with GABAergic neurons constituting 20–40% of this population (Oliver et al., 1994; Winer et al., 1996; Merchán et al., 2005; Mellott et al., 2014). Both glutamatergic and GABAergic IC cells likely receive direct cholinergic inputs. Yigit et al. (2003) provided evidence that cholinergic inputs directly activate GABAergic IC cells during development. Sottile et al. (2017) showed that both GABAergic and glutamatergic IC cells can express nicotinic receptors, but their methods did not provide information on the subcellular localization of those receptors (in fact, their study was focused on cholinergic effects on the axon terminals of IC cells that project to the thalamus). An understanding of cholinergic effects in the IC will require identification of the cell types that receive direct cholinergic inputs.

The major source of cholinergic input into the IC is from the pontomesencephalic tegmentum (PMT; Motts and Schofield, 2009, 2011; Schofield et al., 2011). The PMT is the primary source of cholinergic innervation of the thalamus and brainstem and is closely associated with the sleep-wake cycle, sensory gating and attention (Reese et al., 1995a,b,c; Jones, 2017; Cissé et al., 2018). It comprises two groups of neurons: the laterodorsal tegmental nucleus (LDT) which is situated largely within the periaqueductal gray (PAG), and the pedunculopontine tegmental nucleus (PPT). Of the two components, the PPT is the predominant source of cholinergic inputs to the IC (Motts and Schofield, 2009). At its caudal end, the PPT is ventrolateral to the PAG and surrounds the superior cerebellar peduncle. The PPT extends rostro-ventrally from this location almost as far as the substantia nigra in the rostral and ventral midbrain. Nearly half of the neurons in the PPT region respond to sound, and the cholinergic neurons have been implicated in acoustic startle and tone-specific plasticity (e.g., Reese et al., 1995a,b,c; Xiong et al., 2009; Suga, 2012; Azzopardi et al., 2018).

Here we take advantage of viral vectors and a transgenic rat line to allow for the selective tracing of cholinergic projections into the IC. This is important because the PMT contains a mixed population of neuronal neurotransmitter phenotypes, including cholinergic, GABAergic, and glutamatergic cells (Wang and Morales, 2009). Traditional tract-tracing methods rely on axonal transport of tracers without regard for neurotransmitter phenotype, making it difficult to identify the neurotransmitter associated with any particular axon. We used viral vectors that express fluorescent protein only in cells that contain Cre-recombinase. The vectors were injected into the PPT in ChAT-Cre rats, in which Cre-recombinase is expressed only in cholinergic cells. We then use antibodies against glutamic acid decarboxylase (GAD), a specific marker of GABAergic neurons, to distinguish GABAergic from glutamatergic IC neurons. Our analyses focus on the central nucleus (ICc), the lateral cortex (IClc), and the dorsal cortex (ICd), three of the largest IC subdivisions, and the focus of most previous studies of cholinergic effects in the IC. We observed cholinergic axons from the PPT throughout the IC ipsilateral and contralateral to the labeled PPT cholinergic neurons. The axons typically possessed many boutons, including ones in close apposition to GAD-immunopositive (GAD^+^) and GAD-immunonegative (GAD^−^) neurons in all the IC subdivisions examined. These results suggest that cholinergic axons from the PPT directly contact glutamatergic and GABAergic IC neurons and thus could modulate both excitatory and inhibitory circuits that arise from these cells.

## Materials and Methods

All procedures were conducted following the Northeast Ohio Medical University Institutional Animal Care and Use Committee and National Institutes of Health guidelines. Eighteen Long Evans LE tg (ChAT-Cre) 5.1 Deis rats (Rat Resource and Research Center, University of Missouri; 12 female; six male) received injections of the vector into the PPT. Efforts were made to minimize the number of animals and their suffering. A list of all key resources used in this study are presented in Table 1.

**Table 1:** Key resources.

Reagent type (species) or resource	Designation	Source or Reference	Identifiers	Additional Information
Genetic reagent *(Rattus norvegicus)*	LE-Tg(Chat-Cre)5.1Deis	Rat Resource and Research Center Donor: Dr. Karl Deisseroth (Stanford)	RRRC:00658 RRID: RGD_10401204	
Recombinant DNA reagent	rAAV2/EF1a-DIO-EYFP	UNC Vector Core Donor: Dr. Karl Deisseroth (Stanford)	N/A	titer: 4.6 × 10^12^ http://www.everyvector.com/sequences/show_public/8791
Recombinant DNA reagent	rAAV2/EF1a-DIO-mCherry	UNC Vector Core Donor: Dr. Karl Deisseroth (Stanford)	N/A	titer: 3.2 × 10^12^ http://www.everyvector.com/sequences/show_public/4897
Antibody	anti-GFP (chicken polyclonal)	Thermo Fisher Scientific	Cat#: A10262 RRID: AB_2534023	IHC (1:400)
Antibody	anti-ChAT (goat polyclonal)	Millipore	Cat#: AB144P RRID: AB_2079751	IHC (1:100)
Antibody	anti-GAD67 (mouse monoclonal)	Millipore	Cat#: MAB5406 RRID: AB_2278725	IHC (1:400)
Antibody	anti-NeuN (rabbit polyclonal)	Millipore	Cat#: ABN78 RRID: AB_10807945	IHC (1:500)
Antibody	biotinylated anti-goat (rabbit)	Vector	Cat#: BA-5000 RRID: AB_2336126	IHC (1:100)
Antibody	AF546 streptavidin	Thermo Fisher Scientific	Cat#: S11225 RRID: AB_2532130	IHC (1:100)
Antibody	AF488 streptavidin	Thermo Fisher Scientific	Cat#: S11223 RRID: AB_2336881	IHC (1:100)
Antibody	AF564 anti-mouse (donkey polyclonal)	Thermo Fisher Scientific	Cat#: A10036 RRID: AB_2534012	IHC (1:100)
Antibody	AF750 anti-rabbit (goat)	Thermo Fisher Scientific	Cat#: A21039 RRID: AB_10375716	IHC (1:100)
Software	Neurolucida	MBF Bioscience	RRID: SCR_001775	

### Surgery

Each rat was deeply anesthetized with isoflurane in oxygen (3.5%–5% isoflurane for induction; 1.75%–3% for maintenance). The rat’s head was shaved and disinfected with Betadine (Perdue Products L.P., Stamford, CT, USA). Atropine sulfate (0.08 mg/kg, i.m.) was given to minimize respiratory secretions and Ketofen (ketoprofen; 5 mg/kg, s.c.; Henry Schein, Melville, NY, USA) or Meloxicam SR (1.5 mg/kg, s.c.; ZooPharm, Laramie, WY, USA) was given for pain management. Moisture Eyes PM ophthalmic ointment (Bausch and Lomb, Rochester, NY, USA) was applied to each eye to protect the cornea. The animal’s head was positioned in a stereotaxic frame with a mouth bar positioned 3.5 mm ventral to the horizontal plane through interaural zero. Body temperature was maintained with a feedback-controlled heating pad. Sterile instruments and aseptic techniques were used for all surgical procedures. An incision was made in the scalp and the surrounding skin was injected with 0.5% bupivacaine (Hospira, Inc., Lake Forest, IL, USA), a long-lasting local anesthetic. A craniotomy was made using a dental drill. A 1 μl Hamilton microsyringe was mounted in a manipulator that was rotated caudally in the sagittal plane so that the syringe came in at a 30° angle above the horizontal axis. Following viral injection, Gelfoam (Harvard Apparatus, Holliston, MA, USA) was placed in the craniotomy and the scalp sutured. The animal was then removed from the stereotaxic frame and placed in a clean cage. The animal was monitored until it could walk, eat, and drink without difficulty.

### Viral Tracing

Long Evans LE tg (ChAT-Cre) 5.1 Deis rats were obtained from the Rat Resource and Research Center (University of Missouri). Cre-recombinase is expressed in nearly all cholinergic neurons in these animals (Witten et al., 2011). Two viral vectors were used. Each vector delivers a gene for the expression of fluorescent protein (EYFP or mCherry). The gene is in double-inverted orientation (DIO), so it is expressed only in neurons that contain Cre-recombinase (i.e., in cholinergic neurons). rAAV2/EF1a-DIO-EYFP (titer: 4.6 × 10^12^; UNC Vector Core) or rAAV2/EF1a-DIO-mCherry (titer: 3.2 × 10^12^; UNC Vector Core) was injected in the right PPT of each animal. Coordinates for the injections were chosen to target the caudal PPT, where the cholinergic cells that project to the IC are concentrated (Motts and Schofield, 2009). In two animals, 50 nl vectors were deposited over 2 min at a single site. In the remaining animals, 300–400 nl was delivered over 10 min at one site (12 animals) or each of two sites (four animals). In the latter cases, the syringe was inserted twice, so that one deposit was positioned 0.4–0.5 mm dorsal to the other. After each deposit, the syringe was left in place for 2 min before being withdrawn.

### Perfusion and Tissue Processing

Four weeks after surgery, the animal was deeply anesthetized with isoflurane and perfused transcardially with Tyrode’s solution, followed by 250 ml of 4% paraformaldehyde in 0.1 M phosphate buffer, pH 7.4 and then by 250 ml of the same fixative with 10% sucrose. The brain was removed and stored at 4°C in fixative with 25–30% sucrose for cryoprotection. The following day, the brain was prepared for processing by removing the cerebellum and cortex and blocking the remaining piece with transverse cuts posterior to the cochlear nucleus and anterior to the medial geniculate body. The tissue was frozen and cut on a sliding microtome into 40 μm thick transverse sections, collected in six sets. Before staining, sections were permeablized in 0.2% Triton X-100 in phosphate-buffered saline (PBS) for 30 min at room temperature, then blocked in 10% normal goat serum in 0.2% Triton X-100 and PBS for 1 h, also at room temperature. Sections were then processed for markers as described below. EYFP label was amplified using an antibody against the green fluorescent protein (GFP, 1:400, Molecular Probes A10262; RRID: AB_2534023; note this antibody cross-reacts with EYFP) in combination with a Tyramide Signal Amplification Kit (Molecular Probes). In two cases, an antibody against ChAT (Chemicon AB144P 1:100; RRID: AB_2079751) was used to verify that viral expression was limited to cholinergic neurons. Putative GABAergic cells were stained with an antibody against GAD67 (1:400; Millipore MAB5406; RRID: AB_2278725). Neurons were counterstained with an antibody against the neuronal nuclear protein (NeuN; 1:500; Millipore ABN78; RRID: AB_10807945). In cases where the ChAT antibody was used, a biotinylated anti-goat antibody (1:100; Vector BA-5000; AB_2336126) was used followed by an AF546 Streptavidin tag (1:100; Thermo Fisher Scientific Cat# S-11225; RRID: AB_2532130) to label ChAT-positive neurons in the PPT. In all other cases, a mixture of an AF488 streptavidin tag (1:100; Thermo Fisher Scientific Cat# S11223; RRID: AB_2336881), an AF564 conjugated anti-mouse secondary (1:100; Thermo Fisher Scientific Cat# A10036, RRID: AB_2534012), and an AF750 conjugated anti-rabbit secondary (1:100; Thermo Fisher Scientific Cat# A21039; RRID: AB_10375716) were used to label GFP, GAD67, and NeuN, respectively. Stained sections were mounted on gelatin-coated slides, allowed to dry and coverslipped with DPX.

### Data Analysis

Cholinergic neurons stained with anti-ChAT or labeled by the viral vector injections were used to identify the cholinergic nuclei according to previously published criteria (Motts et al., 2008). In the original study describing the generation of the ChAT-Cre rats, the authors tested the specificity of labeling after injection of a viral vector carrying a gene for Cre-dependent expression of YFP (Witten et al., 2011). Their results showed that over 90% of the YFP-labeled neurons were immunoreactive for ChAT, a specific marker of cholinergic neurons. To ensure that subsequent mutations had not interfered with that specificity, we immunostained sections from two animals with anti-ChAT. IC subdivisions were also identified based on previous criteria (Coote and Rees, 2008; Beebe et al., 2016). Plots and analyses were performed using a Neurolucida system (MBF Bioscience; RRID: SCR_001775) attached to a Zeiss AxioImager Z2 fluorescence microscope (Carl Zeiss MicroImaging, Inc., Thornwood, NY, USA).

For each experiment, fluorescent neurons in the PMT were plotted to visualize the extent of the injection site. The IC was then examined at high magnification in every sixth section through its entire rostral-caudal extent (typically 4–6 sections per animal). The amount of labeling varied across animals. In some cases, especially those with fewer labeled cells in the PPT, there were no labeled axons in the IC; this was not unexpected, given that PPT cholinergic cells projects to many structures throughout the brainstem and thalamus. Those cases were excluded from the present study. Labeled axons were examined to assess possible routes from the PPT to the IC. The results were consistent across cases and suggested several possible routes to both ipsilateral and contralateral IC. To illustrate routes to and within the IC, a representative case with many labeled axons was chosen for detailed plotting. The labeled axons were drawn in every sixth section through the midbrain. The distribution of boutons within the IC was also documented by visual examination of every sixth section through the ipsilateral and contralateral IC. The overall pattern of labeling was consistent across cases, with variations appearing to relate generally to the relative size of the injections (i.e., the relative number of labeled cells in the PMT). For a quantitative description of the labeled boutons, we selected two cases with a large number of labeled axons. We then plotted the labeled boutons, identified as distinct swellings along a labeled axon, in every sixth section through the IC.

A substantial number of labeled boutons appeared to be in close apposition to an IC neuron that was labeled with anti-NeuN and, in some cases, anti-GAD67. Such contacts were apparent across animals; two with a large number of labeled axons and robust immunostaining for both NeuN and GAD were chosen for quantitative analysis. Sections spaced 240 μm apart were chosen to include large parts of the major IC subdivisions in each animal (two sections from one case and three from the second case). The IC in each section was examined at high magnification (63× objective, NA 1.4) and the location of each neuron that appeared to be contacted by a cholinergic axon was plotted with a symbol indicating whether the neuron was GAD^−^ or GAD^+^.

Photomicrographs were taken with a Zeiss AxioImager Z2 fluorescence microscope with either an AxioCam HRm camera (Zeiss) or an Orca Flash 4.0 camera (Hamamatsu). Additions of color, scale bars, and arrows as well as, cropping and global adjustment of levels were done in Adobe Photoshop (Adobe Systems).

## Results

### Injection of Viral Vector Into PPT Labels Cholinergic Neurons

**Figure 1:**
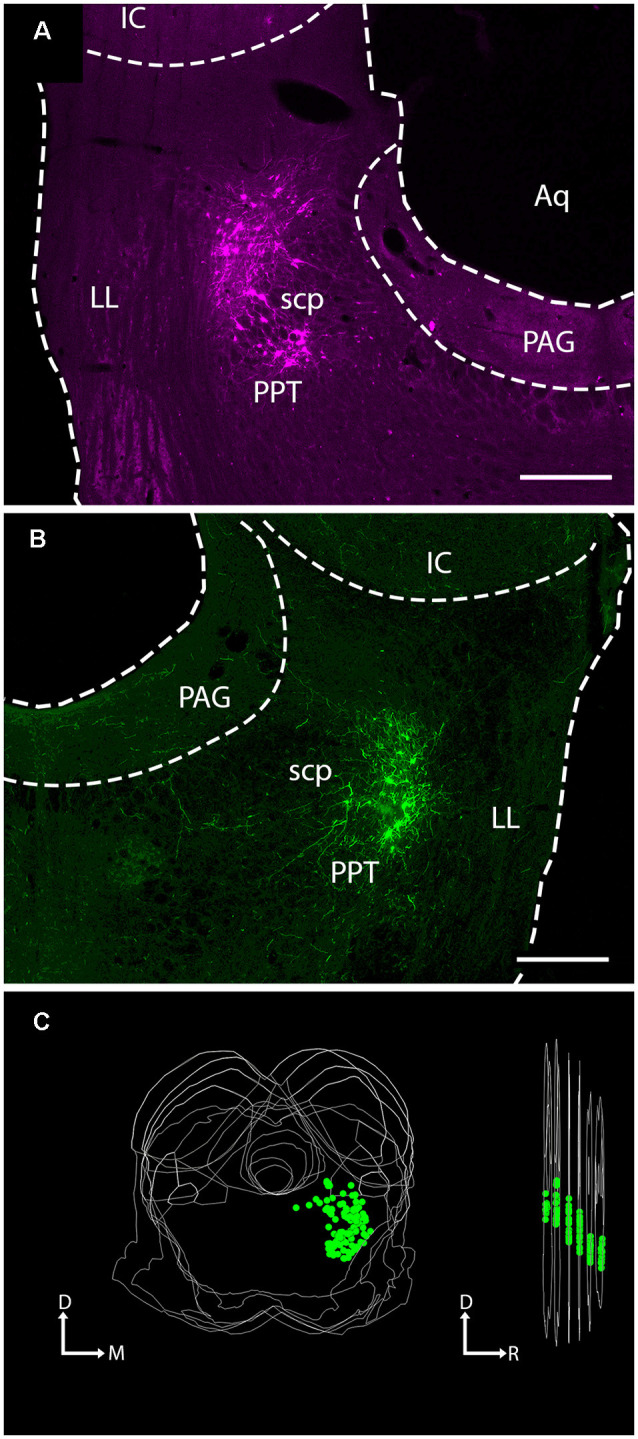
Injection of viral vector into the PPT of ChAT-Cre transgenic rats labeled neurons at the site of injection. Photomicrographs show virally-expressed fluorescent protein in PPT neurons. **(A)** A representative example of mCherry expression in neurons of the left PPT following injection of rAAV2/EF1a-DIO-mCherry. Transverse section; lateral is left, dorsal is up. Scale bar = 500 μm. **(B)** A representative example of EYFP expression in neurons of the right PPT following injection of rAAV2/EF1a-DIO-EYFP. Transverse section; lateral is right, dorsal is up. Scale bar = 500 μm. **(C)** A representative three-dimensional reconstruction showing the extent of labeled neurons in the PPT through six transverse sections (spacing: 240 μm between sections). The image on the left shows the six sections stacked, with each EYFP-labeled cell indicated by a green circle. The image on the right shows the stack rotated for a lateral view. The “cloud” of labeled cells shifts ventrally moving from caudal to rostral, reflecting the orientation of the PPT. Aq, cerebral aqueduct; D, dorsal; IC, inferior colliculus; LL, lateral lemniscus; M, medial; PAG, periaqueductal gray; PPT, pedunculopontine tegmental nucleus; R, rostral, scp, superior cerebellar peduncle.

Injection of either AAV-EF1a-DIO-mCherry or AAV-EF1a-DIO-EYFP into the PPT of Long Evans ChAT-Cre transgenic rats yielded expression of fluorescent protein in neurons associated with the PPT (Figure 1). The number of labeled cells varied across cases, leading to quantitative differences, but qualitatively the results were similar with the two vectors and across sexes. In one case, we saw labeled neurons in the adjacent LDT, the other component nucleus of PMT (not shown). Results, in this case, did not differ from cases in which the label was confined to PPT. In some cases, additional labeled cells were present in the parabigeminal nucleus, a nucleus on the lateral edge of the rostral midbrain that includes a dense cluster of cholinergic cells. These cases were excluded from the analysis in the present study. In the remaining 18 cases, all produced labeled axons in the IC. Twelve of these cases had substantial labeling (“good to very good”) while the remaining cases had fewer labeled axons that served to support and confirm the conclusions. By plotting the labeled cells in every 6th section, we could assess both the viral spread and the efficacy of the labeling. In the twelve better cases, the labeled cell bodies were located across 1–5 sections, indicating that the injection site extended rostrocaudally from a minimum of less than 240 μm to a maximum of ~1,200 μm. The number of fluorescent cells in these cases ranged from 7 (in a single section) to 328 across five sections. Interestingly, the cases that yielded the most labeled axons in the IC were not those with the most labeled PMT cells; we believe this reflects the fact that only a subset of PMT cells projects to the IC, and these cells are interspersed with those that project to other targets (see Motts and Schofield, 2009; Motts and Schofield, 2010).

Within the PPT, our injections labeled cells mostly in the caudal portion of the nucleus, surrounding the superior cerebellar peduncle at the same rostrocaudal levels as the IC. An example of a large injection site is shown in Figure 1C, where each green marker represents a single EYFP-labeled PPT neuron. Cholinergic neurons are not as densely packed in more rostral regions of the nucleus (which extends as far as the substantia nigra in the ventral midbrain; Mesulam et al., 1983). Our cases contained few or no labeled cells in these rostral regions, so we may have missed a portion of the cholinergic projections to the IC. If so, it is likely to be a very small component because the majority of PPT cells that project to the IC are concentrated in the caudal PPT (Motts and Schofield, 2009).

**Figure 2:**
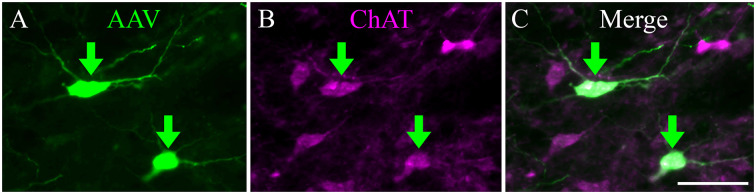
Viral injection selectively labeled ChAT-positive neurons in the PPT. **(A)** EYFP-expressing PPT neurons (green arrows) following AAV injection. **(B)** Same region as shown in **(A)**, but imaged to show immunostaining for ChAT. **(C)** Merged image showing colocalization of ChAT antibody and viral EYFP expression (green arrows). Scale = 100 μm.

We stained sections with antibodies against ChAT to determine whether the expression of the fluorescent protein was limited to cholinergic (i.e., ChAT^+^) cells (Figure 2). In these sections, all virally-labeled PPT neurons were co-labeled with the ChAT antibody (Figure 2, green arrows), indicating that the viral vector is selective for cholinergic neurons. However, it was common to see ChAT^+^ neurons that were not labeled by the viral gene, even though adjacent neurons were so labeled (Figure 2). It is impossible to determine whether this was a failure of the transgene (i.e., Cre-recombinase was not expressed in the cholinergic neuron) or a failure of viral uptake of fluorescent gene expression by the presumptive cholinergic neurons. We chose three cases that produced the most labeled axons in the IC and counted both the virally-labeled cells and the ChAT^+^ PPT cells in the same sections. On average, 18% of the ChAT^+^ cells were labeled by the viral vector (range: 14–23%), suggesting that the efficacy of viral labeling is limited. We completed a similar analysis for a fourth case that had fewer labeled axons in the IC despite having many more labeled cells in the PPT. This case had 155 virally-labeled cells in five sections, which constituted 72% of the ChAT^+^ cells. Clearly, some cases had greater efficacy of labeling, although we never observed 100% labeling. We conclude that the labeled axons and boutons that we observed in the IC are cholinergic and likely underrepresent the PPT-to-IC cholinergic pathway.

### Cholinergic Axons Course Through the Tegmentum to Reach Ipsilateral and Contralateral IC

**Figure 3:**
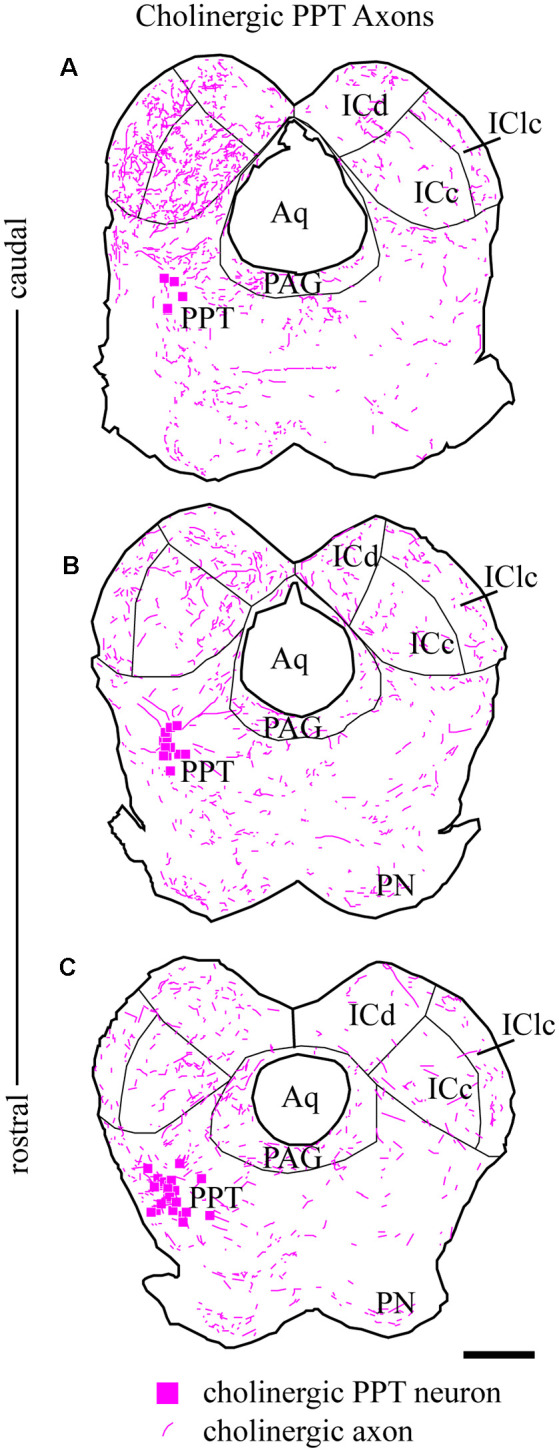
Cholinergic axons travel through the tegmentum to reach ipsilateral and contralateral IC. Fluorescent-labeled axons (magenta lines) were observed throughout transverse IC sections after labeling cholinergic cells in the left PPT (magenta squares). The plots show the axons in a series of transverse sections through the IC at caudal **(A)**, middle **(B)**, and rostral levels **(C)**. On both sides, labeled axons appear to enter the IC all along its ventral border. Sections are 40 μm thick and 240 μm apart. Scale bar = 1 mm.

From the PPT, cholinergic axons travel to many regions of the brainstem and thalamus. Labeled axons were present in the IC bilaterally, with more axons present on the ipsilateral side (Figure 3). Axons coursing toward the IC take multiple routes. Axons leave the PPT dorsally and dorsolaterally to enter the ICc through its ventral border. Axons reaching the ipsilateral IClc do so either through the ventrolateral border of the IC or by coursing through the ICc and turning laterally. Axons traveling to the ICd travel first through either the ICc or the IClc.

The organization of labeled axons suggests several possible routes from the PPT to the contralateral IC. First, axon fragments could be followed from the PPT across the midline, traveling ventral to the PAG or even through the ventral PAG to the ventral border of the contralateral IC. At this point, the axons entered the contralateral IC all along its ventral border and were distributed to each major subdivision in a pattern similar to that in the ipsilateral IC. Also, labeled axons were present in the IC commissure. The directionality of these axons could not be determined, so they could represent a projection from one PPT through the ipsilateral IC to the contralateral IC, or a recurrent loop from the PPT to the contralateral IC and then back to the ipsilateral side.

### Cholinergic Boutons Are Present in all IC Subdivisions

**Figure 4:**
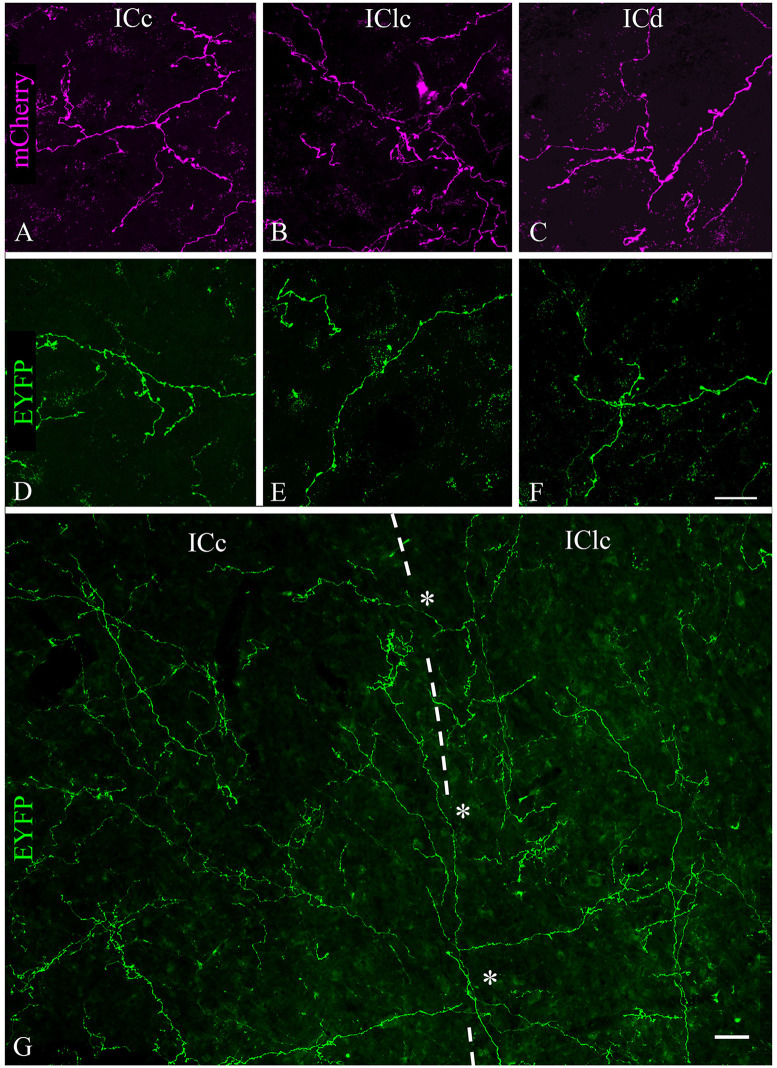
Labeled cholinergic axons with many boutons were present in each subdivision of the IC. **(A–C)** Expression of mCherry in axons from PPT in **(A)** ICc, **(B)** IClc, and **(C)** ICd. **(D–F)** Expression of EYFP in axons from PPT in **(D)** ICc, **(E)** IClc, and **(F)** ICd. Scale bar in **(F)** = 25 μm and applies to **(A–F)**. **(G)** Montage showing EYFP-labeled cholinergic axons in the ICc and IClc (separated by the dashed line), including individual axons that cross the border (asterisks), providing boutons to both subdivisions. Scale = 25 μm.

Labeled axons typically were thin and studded with en passant and terminal boutons. Figure 4 shows results from a case that had substantial labeling of axons in the IC. Cases with fewer labeled axons had fewer boutons but otherwise were similar to one another. In all cases, more boutons were present ipsilaterally than contralaterally and typically were present in all the IC subdivisions on both sides. Individual axons were observed to cross any of the borders between IC subdivisions, with boutons clearly visible in each subdivision; Figure 4G (asterisks) shows examples of axons crossing the ICc/IClc border. Within a subdivision, there was no obvious relationship between the axons or boutons and other features of the subdivision architecture. In the ICc, labeled axons were oriented in several directions without any clear relationship to the orientation of fibrodendritic laminae. In the shell areas (ICd and IClc), the labeled axons showed no particular relationship to borders between layers or between the “GABA modules” and the extramodular domains (see Chernock et al., 2004).

**Figure 5:**
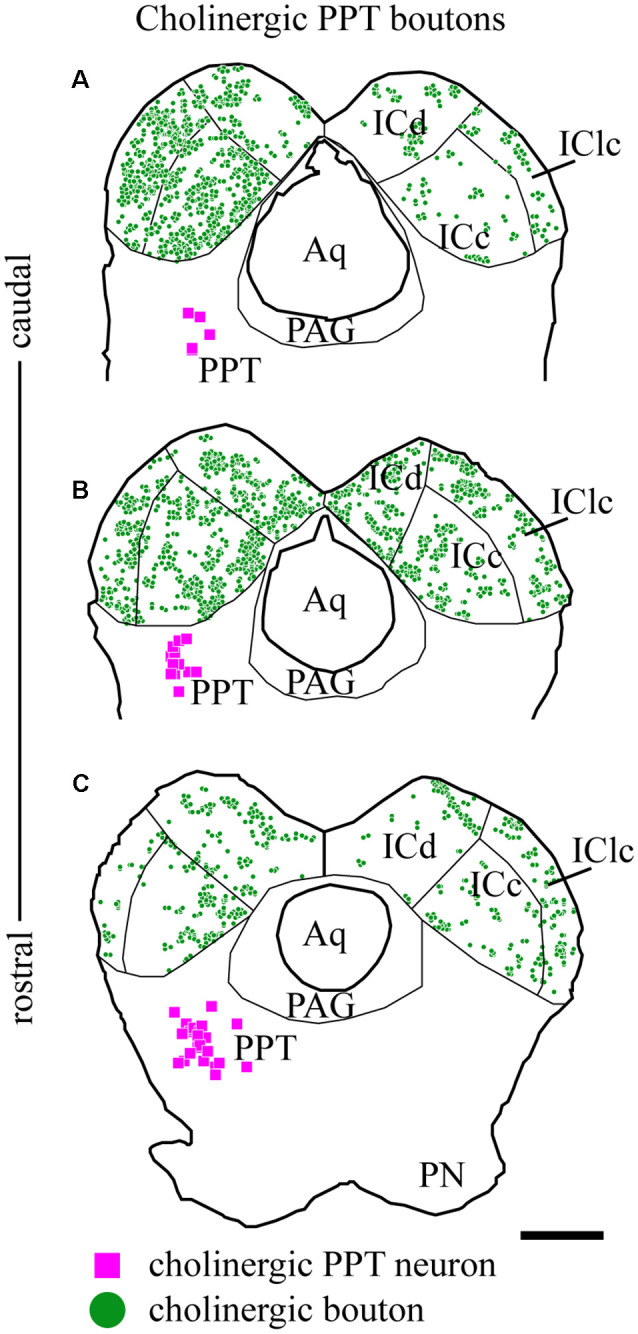
Cholinergic boutons are present in multiple IC subdivisions. Plots of transverse sections through the IC (the same sections illustrated in Figure 2) showing the distribution of labeled boutons (green circles) in the IC after fluorescent labeling of cholinergic cells (magenta squares) in the left PPT. Cholinergic boutons were found in **(A)** caudal, **(B)** mid-IC, and **(C)** rostral sections of IC, where they terminated in the three major subdivisions: dorsal cortex (ICd), lateral cortex (IClc) and central nucleus (ICc). Scale bar = 1 mm.

As described above, the labeled axons in the IC were typically studded with boutons, suggesting many sites of ACh release. Figure 5 plots the distribution of labeled boutons (green circles), demonstrating their wide distribution throughout the ipsilateral and contralateral IC. Consistent with the axonal pattern, the boutons were more numerous on the ipsilateral side than on the contralateral side. Also, labeled boutons were more numerous in the caudal part of the IC, even within a subdivision (compare Figure 5C vs. Figure 5A).

### Cholinergic PPT Axons Contact GAD^+^ and GAD^−^ Neurons in the IC

**Figure 6:**
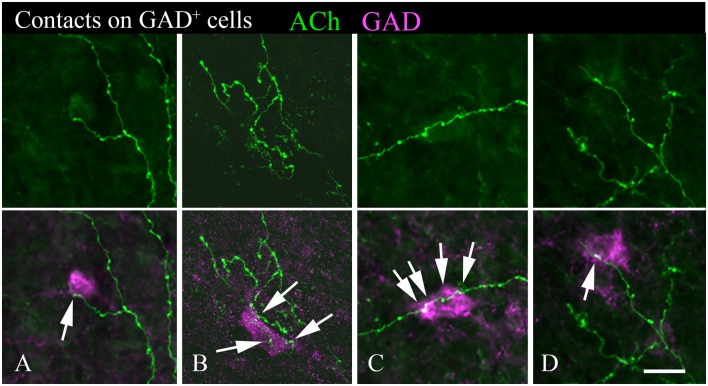
Cholinergic axons contact GAD^+^ neurons in the IC. The upper panel in each column shows fluorescent-labeled cholinergic axons in the IC (green). The lower panel in each column shows the same axons overlaid with an image of the GAD stain (magenta). Arrows show points of close contact between labeled boutons and the GAD^+^ cells. Images **(A,C,D)** are from the IC central nucleus; **(B)** is from IC lateral cortex. Scale bar = 20 μm.

**Figure 7:**
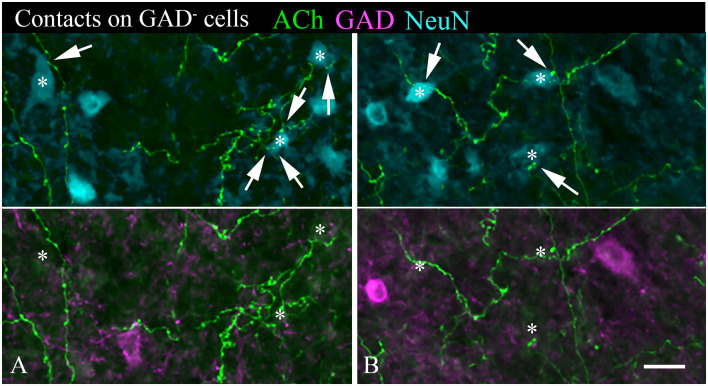
**(A,B)** Cholinergic axons contact GAD^−^ neurons in the IC. The upper panel in each column shows fluorescent-labeled cholinergic axons (green) and NeuN^+^ neurons (cyan). Labeled boutons are in close contact (arrows) with several of the NeuN^+^ cells (*). That these contacted cells are non-GABAergic is shown by the lower panel, which shows GAD immunostain in magenta. None of the asterisk-marked cells are GAD^+^, despite the presence of GAD^+^ cells nearby. IC, lateral cortex. Scale bar = 20 μm.

A majority of the labeled boutons were located in the neuropil between the labeled neuronal cell bodies, but a substantial number of boutons were in close apposition to the cell bodies, suggesting possible synaptic contacts. By staining the tissue with an antibody to GAD, we could examine the relationship of the labeled cholinergic boutons to presumptive GABAergic cells. Figure 6 shows examples of virally-labeled cholinergic boutons (green) in close contact (arrows) with GAD^+^ IC neurons (magenta). We also stained the tissue with a neuron-specific marker (NeuN), allowing us to distinguish GAD^+^ cells from GAD^−^ cells (presumptive glutamatergic neurons). A neuron that is NeuN^+^ and GAD^−^ located close to GAD^+^ profiles is considered to be non-GABAergic. GAD^−^ neurons are likely glutamatergic neurons as IC neurons are either GABAergic or glutamatergic (reviewed by Schofield and Beebe, 2019). Glutamatergic neurons make up the majority of IC neurons and were frequently contacted by labeled cholinergic PPT boutons (Figures 7A,B, arrows).

**Figure 8:**
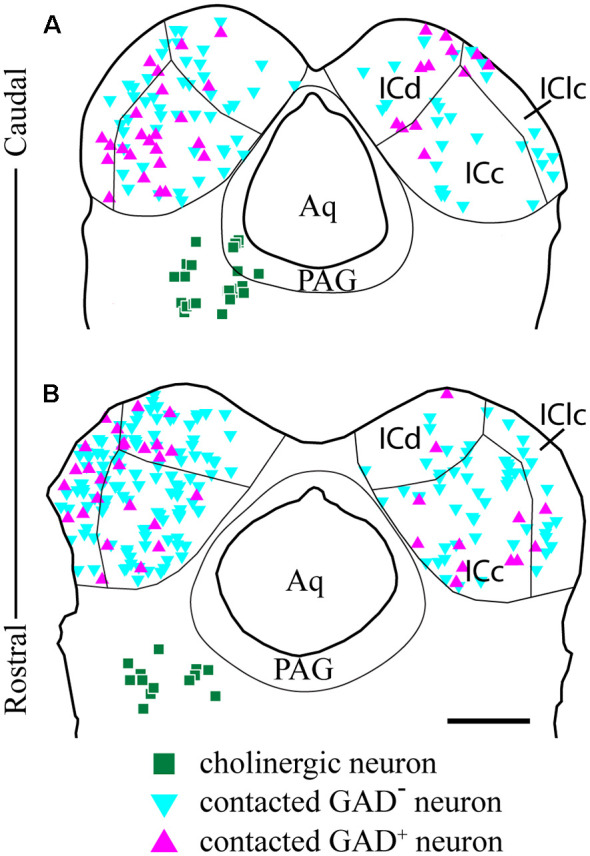
GAD-positive and GAD-negative IC neurons are contacted throughout the IC. Plots of transverse sections through the caudal **(A)** and rostral **(B)** IC show the distribution of neurons contacted by cholinergic boutons after labeling cholinergic cells in the left PPT and laterodorsal tegmental nucleus (LDT; green squares). Contacted cells included both GAD^+^ neurons (magenta triangles) and GAD^−^ neurons (cyan triangles) in each of the major IC subdivisions. Contacted IC neurons were more numerous on the ipsilateral side than the contralateral side. Contacted GAD^−^ neurons outnumbered contacted GAD^+^ neurons. Scale bar = 1 mm.

We observed cholinergic contacts onto neurons in both the ipsilateral and contralateral IC. Figure 8 shows the distribution of contacted cell bodies in two sections through the IC in a representative case. Several points are clear. First, contacts occur bilaterally in all three IC subdivisions, with more contacts ipsilateral than contralateral. Second, contacts occurred on both GAD^−^ cells (presumptive glutamatergic cells, Figure 8, cyan symbols) and GAD^+^ cells (presumptive GABAergic cells, Figure 8, magenta symbols). Thus, cholinergic axons from a single PPT appear to contact GAD^+^ and GAD^−^ neurons in each major IC subdivision both ipsilateral and contralateral to the injected PPT.

### Individual Cholinergic Axons Can Contact Both GAD^+^ and GAD^−^ Neurons

**Figure 9:**
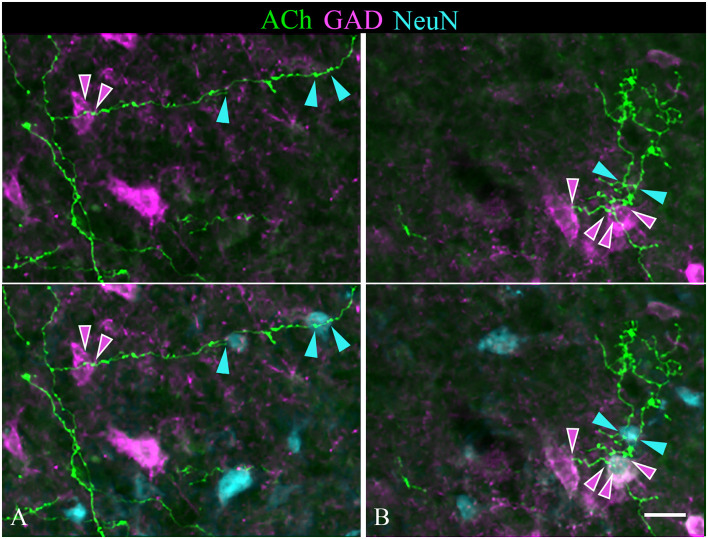
Single cholinergic axons contact both GAD^+^ and GAD^−^ IC neurons. The upper panel in each column shows fluorescent-labeled cholinergic axons (green) overlaid with an image of GAD staining (magenta). The lower panel in each column shows the same image overlaid with an image of the NeuN stain (cyan). **(A)** An axon segment in the IC lateral cortex appears to contact one GAD^+^ neuron (magenta arrowheads) and two different GAD^−^ neurons (cyan arrowheads). **(B)** shows an axon segment from the IC central nucleus that appears to contact two GAD^+^ neurons (magenta arrowheads) and a nearby GAD^−^ neuron (cyan arrowheads). Scale bar = 20 μm.

Individual sections often contained relatively long segments of labeled axons that allowed several observations. In each of the IC subdivisions, individual axons appeared to contact multiple IC neurons. Figure 7 includes examples of single cholinergic axons that appear to contact multiple GAD^−^ neurons. Figure 9 shows additional patterns of multiple targets, including multiple GAD^+^ neurons (Figure 9B) and axons that contact both GAD^+^ and GAD^−^ neurons (Figures 9A,B). Each pattern of contact—onto multiple GAD^+^ neurons, multiple GAD^−^ neurons, or both GAD^+^ and GAD^−^ neurons—was observed ipsilateral and, less often, contralateral to the injection site.

## Discussion

**Figure 10:**
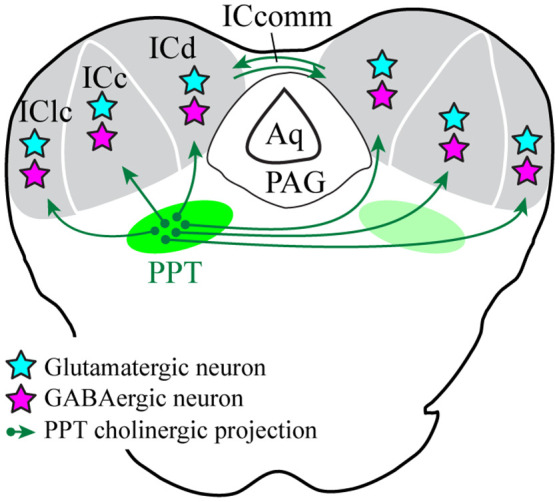
Cholinergic PPT cells project throughout the IC and contact GABAergic and presumptive glutamatergic cells. Green arrows show cholinergic projections from PPT to all three subdivisions of the IC. Cyan stars represent cholinergic-contacted glutamatergic neurons and magenta stars represent cholinergic-contacted GABAergic neurons. Aq, cerebral aqueduct; ICc, d, lc, inferior colliculus central nucleus, dorsal cortex, and lateral cortex; ICcomm, commissure of the IC; PAG, periaqueductal gray; PPT, pedunculopontine tegmental nucleus.

We used selective viral tract-tracing of cholinergic PPT neurons to identify cholinergic projections to the IC. The PPT is a prominent source of cholinergic innervation of the thalamus and brainstem and is involved in wide-ranging functions such as arousal, sensory gating, sleep-wake cycle, and plasticity (reviewed by Schofield et al., 2011; Schofield and Hurley, 2018). The present results demonstrate that cholinergic axons from the PPT terminate in the three largest IC subdivisions: the ICc, ICd, and IClc (Figure 10). These subdivisions contribute to different aspects of hearing, each of which may be affected by cholinergic modulation. The cholinergic axons typically contain many boutons and can cross borders between IC subdivisions as well as within subdivisions (e.g., the laminar boundaries in the IClc). Cholinergic axons contact IC cells that are GAD^+^ (presumptive GABAergic) and GAD^−^ (presumptive glutamatergic), suggesting that ACh acts on both excitatory and inhibitory IC circuits. Moreover, an individual cholinergic axon can contact cells of both neurotransmitter phenotypes. Taken together, PPT cholinergic neurons appear to contact many excitatory and inhibitory cells across multiple IC subdivisions, suggesting wide-ranging effects of ACh in the IC.

### Technical Issues

The combination of Cre-expressing cholinergic cells in transgenic animals and Cre-dependent expression of fluorescent proteins delivered *via* viral vectors provides an opportunity for highly selective labeling of a cholinergic pathway (e.g., Stornetta et al., 2013). Following immunostaining with anti-ChAT, we found that fluorescent protein expression was limited to ChAT^+^ cells. We conclude that the labeled axons were cholinergic. The same analysis showed numerous ChAT^+^ cells that did not express fluorescent protein despite being among other cells that were so labeled. It is unclear whether this reflects a failure of Cre expression or failure of viral uptake and subsequent expression of the fluorescent label. The two vectors used here were serotype 2, selected because of relatively high efficiency in anterograde labeling of neuronal pathways (Aschauer et al., 2013; Salegio et al., 2013). However, possibly a different serotype would label some of the cholinergic cells that were unlabeled here. We conclude that our experiments probably labeled <100% of the pathway of interest, and cannot rule out the possibility that a specific subtype of cholinergic cell failed to express the fluorescent proteins.

There are disparate views on volume vs. synaptic cholinergic transmission (Descarries et al., 1997; Zoli et al., 1999; Parikh et al., 2007; Lendvai and Vizi, 2008; Sarter et al., 2009; Muñoz and Rudy, 2014; Takács et al., 2018), with no data to argue strongly for one mode or the other in the IC. Of course, the specificity of cholinergic action also depends on the nature and location of cholinergic receptors, with opportunities for both presynaptic and postsynaptic effects. As discussed above (Introduction), the IC contains a variety of nicotinic and muscarinic receptor types, but as yet little is known about the specific cells and circuits associated with these receptors. By using light microscopy, we have been able to assess the distribution of cholinergic axons and likely release sites over a large area. The results suggest that a single PPT releases ACh across a wide expanse of both ipsilateral and contralateral IC. Although a majority of boutons were located in the neuropil, many of the cholinergic boutons were in close apposition to neuronal (NeuN^+^) cell bodies, allowing us to assess some of the cell types most likely affected by ACh. By adding immunostain for NeuN and GAD67, we were able to conclude that ACh is likely to have direct effects on both GAD^+^ (likely GABAergic) and GAD^−^ neurons. Given that IC neurons are mostly either GABAergic or glutamatergic, the GAD^−^ neurons are likely to be glutamatergic cells. Our conclusion that ACh affects both glutamatergic and GABAergic IC cells is consistent with previous studies showing physiologic activation of GABAergic cells (Yigit et al., 2003) and the presence of cholinergic receptor mRNA in both cell types (e.g., Sottile et al., 2017). Ultimately, electron microscopy will be needed to identify synaptic release sites for ACh. The present results indicate that such studies will be needed in each of the IC subdivisions.

### Functional Implications

The present results extend the conclusions reached *via* retrograde tracing experiments that identified the PPT as the largest source of cholinergic input to the IC (Motts and Schofield, 2009). Those experiments were based on large injections of tracer that typically encroached on multiple IC subdivisions. The present results show that cholinergic PPT axons innervate each of the large IC subdivisions: ICc, ICd, and IClc. Also, the retrograde tracing studies indicated that, overall, more PPT cells project to the ipsilateral IC and fewer project to the contralateral IC. The present results indicate that the axonal distribution reflects a similar pattern, with denser projections to the ipsilateral IC than contralateral IC. Ipsilateral dominance applies to each of the IC subdivisions.

The widespread distribution of PPT cholinergic axons is consistent with studies of cholinergic receptors, which describe nicotinic and muscarinic receptors throughout the IC (Schwartz, 1986; Glendenning and Baker, 1988; Morley and Happe, 2000; Gahring et al., 2004; Happe and Morley, 2004). Given that cholinergic axons are distributed widely in the IC, one would expect that a majority of IC cells are affected by cholinergic inputs. The available studies are consistent with such a view, but the data are limited to studies in which the location of recorded units was not related to IC subdivision (Curtis and Koizumi, 1961; Watanabe and Simada, 1973; Farley et al., 1983), or to studies in which the units were restricted to the ICc (Habbicht and Vater, 1996; Ji et al., 2001; Yigit et al., 2003). The present results suggest that direct cholinergic effects would be observed upon recording neurons in the ICd and the IClc.

#### Cholinergic Effects in the ICc

Information on cholinergic effects within specific IC subdivisions has been limited to the ICc. Previous studies showed that cholinergic agents affect the firing rate but not the temporal response patterns of IC neuronal responses to sounds (Farley et al., 1983; Habbicht and Vater, 1996). They suggested that ACh acted through nicotinic and muscarinic receptors to set the level of neuronal activity in the IC, likely modulating neuronal sensitivity as well as gain. Cholinergic effects in other subcortical auditory nuclei have led to similar conclusions. Oertel and colleagues (Fujino and Oertel, 2001; Oertel and Fujino, 2001; Oertel et al., 2011) suggested that ACh enhances responses of T stellate cells in the cochlear nucleus, perhaps to enhance coding of spectral peaks and thus to improve interpretation of sounds in noise. Caspary and colleagues have suggested that cholinergic effects in auditory thalamus similarly support hearing in a noisy environment, and further that deterioration of the cholinergic system with aging could relate to presbycusis (Sottile et al., 2017). It is worth noting that the PPT is a source of cholinergic innervation to the cochlear nucleus and auditory thalamus, as well as the inferior colliculus. Moreover, individual PPT cells can send branching axons to innervate two or more of these target structures (e.g., one IC as well as left and right auditory thalamus; Motts and Schofield, 2011). Such divergent projections are typical of many modulatory systems and may reflect a common effect across auditory nuclei (reviewed by Schofield and Hurley, 2018).

The origin of the inputs from the PPT may provide additional insight into cholinergic functions. Within the context of auditory processing, the PPT has been associated with arousal, plasticity (especially driven by top-down circuits) and sensory gating (Xiong et al., 2009; Schofield et al., 2011; Schofield and Hurley, 2018). The PPT projections to the auditory thalamus have been implicated in the enhancement of hearing in a noisy environment, in part by cholinergic enhancement of ascending inhibitory pathways as well as enhancement of top-down modulation (*via* effects on corticothalamic circuits; Sottile et al., 2017). Gut and Winn (2016) proposed that the PPT, particularly the caudal part (as studied here), is especially important for an organism’s ability to act quickly in response to sensory stimuli. This proposal ties together the sensory aspects of the PPT with its well-known ties to the basal ganglia. Many cells in the PPT respond to acoustic stimuli, so it is not surprising that they would contribute to auditory function (Reese et al., 1995a,b,c). Slee and David (2015) have shown that IC cells respond differently depending on whether the subject is performing a task. They concluded that arousal associated with task performance likely accounted for part of the difference in responses. Cholinergic projections as demonstrated in the present study may contribute to such responses. Kuenzel and colleagues (Goyer et al., 2016; Gillet et al., 2018; Kuenzel, 2019) have suggested a similar role for projections from the PPT to the cochlear nucleus, where ACh can modulate neuronal sensitivity in accord with an animal’s behavioral state and level of arousal.

Suga and colleagues have shown evidence for a cholinergic role in corticofugal-driven plasticity of subcortical auditory nuclei, including plasticity in the IC (Xiong et al., 2009; Suga, 2012). Stimulation of the auditory cortex can lead to the retuning of an IC cell’s response selectivity. Such plasticity can involve a variety of stimulus parameters, such as shifting the frequency to which the IC cell responds most readily. The retuning of IC responses can outlast the period of cortical stimulation by minutes to hours as a result of plasticity in both cortical and subcortical circuits. Significantly, that plasticity is dependent on ACh in the IC. We believe that the details of the retuning (e.g., whether a given cell retunes to a higher or a lower acoustic frequency) is determined by the massive direct projections from the auditory cortex to the IC, with cortical axons activating both excitatory and inhibitory circuits within the IC. ACh, on the other hand, provides a permissive signal that allows synaptic plasticity to sustain the effects of the cortical stimulation. Such a permissive signal could: (1) be elicited by direct auditory cortical projections to cholinergic PPT cells that project to the IC (Schofield and Motts, 2009; Schofield, 2010); and (2) affect multiple IC cells *via* highly divergent cholinergic axons, including axons that contact both glutamatergic and GABAergic IC cells (current results).

Interestingly, the work by Suga (2012) like that cited earlier regarding cholinergic effects on IC cells, focused on responses of cells in the ICc. It will be of interest in future studies to identify the effects of ACh on responses of IC cells outside the central nucleus, particularly given these non-central regions being the prime targets of cortical inputs as well as centers for multisensory processing.

#### Cholinergic PPT Axons Likely Modulate Lemniscal and Extralemniscal Auditory Pathways

The termination of cholinergic axons across IC subdivisions is significant when we consider that each IC subdivision contributes differentially to three parallel auditory pathways: a lemniscal pathway, a polysensory pathway, and a diffuse pathway (Calford and Aitkin, 1983; Rouiller, 1997; Mellott et al., 2014). Each pathway is thought to serve different aspects of hearing. The lemniscal pathway is tonotopically organized and provides the primary-like representation of sound. It encompasses projections from the ICc to the ventral medial geniculate nucleus and on to tonotopically organized regions of the auditory cortex. The polysensory pathway incorporates inputs from other sensory systems, especially the somatosensory system. These inputs terminate heavily in the IClc. Projections from the IClc, along with projections from ICc, converge in the medial division of the medial geniculate nucleus, which then projects widely to auditory cortical areas and other forebrain targets to form the polysensory pathway (De Ribaupierre, 1997). Finally, the diffuse pathway originates from the ICd, which projects to the dorsal medial geniculate nucleus and then on to secondary and temporal auditory cortical areas. The ICd has long stood out as unique among IC subdivisions, with descending inputs from the auditory cortex appearing to dominate over ascending inputs (Ehret, 1997). ICd cells are typically broadly-tuned and respond only weakly to simple auditory stimuli, but respond more robustly to complex contextual stimuli (such as the cries of isolated offspring; De Ribaupierre, 1997). Early lesion studies suggested that ICd plays a particularly important role in auditory attention (Jane et al., 1965). The present results show that cholinergic axons from the PPT terminate broadly in each of the large IC subdivisions and are in a position to modulate the activity in each of the ascending parallel pathways. At the level of the thalamus (another target of PPT cholinergic projections), ACh can have different effects on lemniscal vs. extra-lemniscal cells (Mooney et al., 2004). The differences are thought to be mediated through different cholinergic receptors. In the IC, Gahring et al. (2004) noted higher levels of the nicotinic receptor subunit β4 in the IClc compared to other IC subdivisions. Happe and Morley (2004) noted high levels of the nicotinic receptor α7 subunits in the IClc. To the best of our knowledge, none of these receptors have been associated with specific output pathways from the IClc or ICd. Nonetheless, the current data, along with the prominent contributions of each IC subdivision to the parallel ascending pathways, suggests that PPT cholinergic modulation could affect a wide range of auditory functions.

#### Cholinergic Axons Target Both GABAergic and Glutamatergic IC Neurons

Several studies have shown that ACh affects the majority of IC cells’ response to sound (Watanabe and Simada, 1973; Farley et al., 1983; Habbicht and Vater, 1996). The effects can be mediated *via* nicotinic and muscarinic receptors. In general, cholinergic agents can affect response rate but appear to have little effect on the temporal patterns of a neuron’s response to a sound, or on the selectivity for specific stimulus parameters. These studies could not discern direct from indirect cholinergic effects on the recorded cell, so the underlying mechanisms remain unclear. The present study suggests that the cholinergic effects are mediated by direct actions on both excitatory and inhibitory IC cells. We showed that PPT cholinergic axons are closely associated with both glutamatergic and GABAergic IC neurons. This sets the stage for postsynaptic effects on the closely apposed cell bodies or dendrites (without ruling out presynaptic effects on nearby axon terminals that contact the same postsynaptic cell). Evidence for cholinergic effects on identified GABAergic or glutamatergic IC cells is limited. Sottile et al. (2017) showed that GABAergic and presumptive glutamatergic IC cells express mRNA for nicotinic receptor subunits. These results do not indicate where the receptors are expressed on the cells [indeed, Sottile et al. (2017) were examining cholinergic effects on the collicular axon terminals in the thalamus]. *In vitro* recordings from the IC provide additional support. Yigit et al. (2003) provided physiological evidence that GABAergic IC cells are activated *via* muscarinic receptors. Those experiments were conducted in young animals and could potentially reflect mechanisms that disappear after the system has developed (see Morley and Happe, 2000 for a discussion of separate cholinergic roles during and after the development of the auditory system). In a preliminary study, Rivera-Perez et al. (2020) demonstrated direct nicotinic depolarization of VIP-expressing IC cells (which are known to be glutamatergic; Goyer et al., 2019). Additional experiments will be needed to identify the receptor types on glutamatergic and GABAergic IC cells and to differentiate presynaptic vs. postsynaptic effects.

## Conclusion

The PPT, the largest source of cholinergic projections to the IC, sends axons to terminate bilaterally throughout the three major subdivisions of the IC. This termination pattern suggests that ACh modulates auditory processing associated with all three parallel ascending pathways to the thalamus—the tonotopic, multisensory, and diffuse pathways—and thus affects most aspects of auditory processing. Cholinergic boutons are found in close association with both glutamatergic and GABAergic IC cells, suggesting that ACh modulates both excitatory and inhibitory IC circuits. Overall, the PPT is likely to set the sensitivity of IC cells, modulating neuronal responses according to behavioral state and level of arousal. Further, by providing a permissive signal for plasticity in IC cells driven by top-down (corticofugal) mechanisms, ACh is likely to have both long-term as well as short-term effects on midbrain auditory processing.

## Data Availability Statement

The original contributions presented in this study are included in the article. Further inquiries can be directed to the corresponding author.

## Ethics Statement

The animal study was reviewed and approved by Northeast Ohio Medical University Institutional Animal Care and Use Committee.

## Author Contributions

All authors contributed to the conception and design of the study and data collection. WN analyzed the data and wrote the first draft of the manuscript. All authors contributed to manuscript revision, and read and approved the submitted version.

## Conflict of Interest

The authors declare that the research was conducted in the absence of any commercial or financial relationships that could be construed as a potential conflict of interest.
